# Supplementary material for: Brain network coupling associated with cognitive performance varies as a function of a child’s environment in the ABCD study
Source: Nat Commun. 2021 Dec 10;12:7183. doi: 10.1038/s41467-021-27336-y (PMC8664837; doi:10.1038/s41467-021-27336-y)
Supplement: Supplementary file 1 — Supplementary Information [file 41467_2021_27336_MOESM1_ESM.pdf]

Supplementary Information for: "Brain network coupling associated with cognitive performance varies as a function of a child's environment in the ABCD study"

Contents

Supplementary Note 1. Additional demographic and environmental information. 2
Supplementary Table 1 2
Supplementary Note 2. Identification of environmental variables 4
Supplementary Table 2. 5
Supplementary Note 3. Scatterplots relating resting state metrics and cognitive test performance ..7
Supplementary Figure 1 7
Supplementary Note 4. Relations between LFPN-DMN connectivity and cognitive test performance, separated by test 8
Supplementary Figure 2. 8
Supplementary Note 5. Relations between LFPN-LFPN connectivity and cognitive test performance, separated by test 9
Supplementary Note 6. Bootstrapped distribution of LFPN-DMN connectivity ~ test performance parameter estimates..9
Supplementary Figure 3. 10
Supplementary Note 7. Permutation testing of LFPN-DMN connectivity ~ test performance parameter estimates..10
Supplementary Note 8. Relations between LFPN-DMN connectivity and cognitive test performance, for children with low thresholds of motion 11
Supplementary Note 9. Relations between LFPN-DMN connectivity and cognitive test performance, controlling for number of usable frames 11
Supplementary Figure 4. 12
Supplementary Note 10. Relations between LFPN-DMN connectivity and age 12
Supplementary Figure 5. 13
Supplementary Note 11. Ridge regression confidence intervals 13
Supplementary Note 12. Deviations from pre-registration 14
Supplementary Note 13. References..15

Supplementary Note 1. Additional demographic and environmental information.

Supplementary Table 1. Wider environmental information. Variables included in the ridge regression predicting cognitive test scores. All except income were used in primary models; additional tests confirmed that income did not add predictive power above and beyond these variables. *P*-values without correction obtained from two-sided t-tests, calculated using the tableone package in R.

	**Above poverty (*n* = 5805)**	**Below poverty (*n* = 1034)**	**p-test**
Combined family income (%)			<0.001
Less than $5,000	0 (0.0)	187 (18.1)	
$5,000 through 11,999	0 (0.0)	219 (21.2)	
$12,000 through $15,999	0 (0.0)	154 (14.9)	
$16,000 through $24,999	0 (0.0)	280 (27.1)	
$25,000 through $34,999	215 (3.7)	194 (18.8)	
$35,000 through $49,999	579 (10.0)	0 (0.0)	
$50,000 through $74,999	972 (16.7)	0 (0.0)	
$75,000 through $99,999	1050 (18.1)	0 (0.0)	
$100,000 through $199,999	2157 (37.2)	0 (0.0)	
$200,000 and greater	832 (14.3)	0 (0.0)	
Parents' highest level of education (n, %)			<0.001
3rd grade	1 (0.0)	0 (0.0)	
4th grade	0 (0.0)	1 (0.1)	
5th grade	0 (0.0)	1 (0.1)	
6th grade	4 (0.1)	13 (1.3)	
7th grade	1 (0.0)	2 (0.2)	
8th grade	1 (0.0)	8 (0.8)	
9th grade	6 (0.1)	24 (2.3)	
10th grade	10 (0.2)	26 (2.5)	
11th grade	12 (0.2)	34 (3.3)	
12th grade	13 (0.2)	47 (4.5)	
High school graduate	167 (2.9)	169 (16.3)	
GED or equivalent	66 (1.1)	91 (8.8)	
Some college	590 (10.2)	297 (28.7)	
Associate degree: occupational	374 (6.4)	135 (13.1)	
Associate degree: academic	297 (5.1)	63 (6.1)	
Bachelor's degree	1818 (31.3)	86 (8.3)	
Master's degree	1677 (28.9)	32 (3.1)	
Professional school degree	364 (6.3)	4 (0.4)	
Doctoral degree	403 (6.9)	1 (0.1)	
People living in home (mean (SD))	4.76 (1.64)	4.97 (2.89)	0.001

Any siblings (yes, %)	1905 (32.8)	269 (26.0)	<0.001
Hours/week spent at another household (mean (SD))	5.34 (19.45)	5.45 (21.63)	0.869
Financial stress (0-7; mean (SD))	0.28 (0.85)	1.32 (1.61)	<0.001
Race (%)			<0.001
Native American/Alaska Native	17 (0.3)	14 (1.4)	
Asian	126 (2.2)	8 (0.8)	
Black/African American	495 (8.5)	377 (36.5)	
Pacific Islander	8 (0.1)	1 (0.1)	
Other	159 (2.7)	74 (7.2)	
White	4263 (73.4)	386 (37.3)	
Mixed	696 (12.0)	141 (13.6)	
Refuse to answer	41 (0.7)	33 (3.2)	
Hispanic/Latino ethnicity (no, %)	4776 (83.1)	682 (67.3)	<0.001
Parent marital status (%)			<0.001
Married	4621 (79.7)	302 (29.6)	
Widowed	33 (0.6)	22 (2.2)	
Separated/divorced	600 (10.4)	232 (22.7)	
Never married	319 (5.5)	369 (36.1)	
Living with partner	223 (3.8)	96 (9.4)	
Generational status (%)			<0.001
Parent born outside U.S.	708 (12.2)	201 (19.5)	
Grandparent born outside U.S.	933 (16.1)	90 (8.7)	
Child born outside U.S.	118 (2.0)	32 (3.1)	
Parents and grandparents born in U.S.	4043 (69.7)	709 (68.7)	
School setting (%)			<0.001
Not in school	19 (0.3)	6 (0.6)	
Regular public school	4836 (83.3)	891 (86.2)	
Regular private school	346 (6.0)	40 (3.9)	
Charter school	412 (7.1)	79 (7.6)	
Vocational/tech school	2 (0.0)	1 (0.1)	
Cyber school	7 (0.1)	2 (0.2)	
Home school	112 (1.9)	2 (0.2)	
School for behavioral/emotional problems	7 (0.1)	3 (0.3)	
Other	63 (1.1)	10 (1.0)	
Youth-reported supportive school environment (6-24; mean (SD))	19.95 (2.63)	19.96 (3.22)	0.949
Youth-reported school involvement (4-16; mean (SD))	13.11 (2.25)	13.22 (2.44)	0.162
Youth-reported school disengagement (2-8; mean (SD))	3.66 (1.39)	3.79 (1.57)	0.006
Census: % of people over age 25 with at least a high school diploma (mean (SD))	91.13 (8.76)	81.30 (12.11)	<0.001

Census: income disparity (mean (SD))	1.81 (1.17)	3.13 (1.34)	<0.001
Census: % of occupied units without complete plumbing (mean (SD))	0.28 (0.64)	0.44 (0.83)	<0.001
Census: % of families below the poverty level (mean (SD))	8.35 (8.68)	20.93 (14.61)	<0.001
Census: % of labor force aged >=16 y unemployed (mean (SD))	7.69 (4.52)	13.15 (7.49)	<0.001
Census: uniform crime reports (mean (SD))	43774.47 (69634.30)	43204.49 (57108.32)	0.81
Census: adult violent crime reports (mean (SD))	2660.87 (6271.58)	2642.93 (5030.45)	0.933
Census: estimated lead risk (1-10; mean (SD))	4.40 (2.98)	6.77 (2.89)	<0.001
Parent-reported neighborhood safety (1-5; mean (SD))	4.05 (0.85)	3.34 (1.11)	<0.001
Parent self-reported aggressive behavior (0-30; mean (SD))	3.14 (3.27)	4.47 (4.58)	<0.001
Parent self-reported intrusive behavior (0-12; mean (SD))	1.01 (1.43)	1.08 (1.43)	0.198
Parent self-reported withdrawn behavior (0-18; mean (SD))	1.35 (1.85)	2.46 (2.83)	<0.001
Parent ethnic identification (1-5; mean (SD))	2.71 (0.86)	2.58 (0.94)	<0.001
Youth-reported family conflict (0-9; mean (SD))	1.93 (1.92)	2.45 (2.04)	<0.001
Youth-reported parental monitoring (1-5; mean (SD))	4.43 (0.46)	4.31 (0.59)	<0.001
Youth-reported parental acceptance (1-3; mean (SD))	2.80 (0.29)	2.76 (0.33)	<0.001

Supplementary Note 2. Identification of environmental variables

In order to identify environmental variables to include in our ridge regression, we began by identifying all measures which were collected for all families at the baseline timepoint of the ABCD study which may characterize children's environments. This included those relating to demographics, neighborhood, school, parenting, and culture. We did not include items more directly related to the child's behavior, like screen time or substance use, nor items more directly related to family members' health and wellbeing.

In general, we aimed to include each of these measures. However, there were several exceptions, as we also wanted to limit the absolute number of measures:

1. When there were measures that were likely to be measuring the same construct, we chose to retain only those variables which previous literature could theoretically link to children's test performance (e.g., "census: median home value" might be better captured by the family's precise combined income and by other neighborhood measures such as "census: income disparity and census: percentage of families below poverty").
2. Similarly, when the same survey measure of the environment was administered to both parent and child, we chose the child's response over the parent's.
3. When there were multiple variables that could be subsumed under a single summary measure, and we had more reason to believe that this summary was meaningful as opposed to each separate measure, we used the summary measure (e.g., both "parent and parent partner highest level of education" were recoded to indicate "combined highest year of education," as in previous work).

Once we gathered our list of environmental variables, we pre-registered these prior to running any analyses. The purpose of the pre-registration was to ensure that we thought carefully about each variable we selected ahead of time and did not alter the list on the basis of our results. A full table listing each and our use is included below.

Supplementary Table 2. Decisions about environmental variables used in ridge regression.

Identified measure	Included as is	Included as part of summary or recoded	Not included	Not available for all children at baseline
child native language				x
parent native language				x
amount of English spoken to child				x
parent race		x		
other languages (parent)				x
other languages (child)				x
family born outside U.S.		x		
parent marital status	x			
parent highest level of education		x		
parent partner highest level of education		x		
parent employment			x	
parent partner employment			x	
total combined family income	x			
parent income		x		
parent partner income		x		
financial stress	x			
people in home	x			
country child was born		x		
country parent was born		x		
anyone in child's family born not in U.S.		x		
where biological father was born			x	
where biological mother was born			x	
amount of time child spends in other household	x			
other household where child spends a significant amount of time		x		
ethnic identification (MEIM-R) exploration		x		
ethnic identification (MEIM-R) commitment and attachment		x		
ethnic identification (MEIM-R) overall	x			
family environment conflict (youth report)	x			
family environment conflict (parent report)			x	
parental monitoring	x			
parental acceptance subscale	x			
school environment subscale	x			
school involvement subscale	x			
discrimination				x
school setting	x			
total bad life events				x
total good life events				x

sum of negative life events and how much affected				x
sum of positive life events and how much affected				x
number of brothers		x		
number of sisters		x		
twin or triplet		x		
number of younger siblings		x		
number of older siblings		x		
number of half brothers		x		
number of half sisters		x		
any siblings	x			
parent aggressive behavior (ASR)	x			
parent intrusive behavior (ASR)	x			
parent rule break (ASR)			x	
parent withdrawn (ASR)	x			
parent total problems (ASR)			x	
census: area deprivation index			x	
census: crowding			x	
census: percentage of population aged >=25 y with at least a high school diploma	x			
census: percentage of homeowner			x	
census: median home value			x	
census: income disparity	x			
census: median family income			x	
census: median monthly mortgage			x	
census: percentage of occupied housing unites without complete plumbing	x			
census: percentage of families below poverty	x			
census: median gross rent			x	
census: percentage unemployed	x			
census: percentage in white collar positions			x	
census: residential density			x	
census: drug possession			x	
census: drug sale			x	
census: drug abuse violations			x	
census: uniform crime reports	x			
census: lead risk	x			
census: total adult offenses			x	
census: total violent crimes	x			
census: walkability			x	

Supplementary Note 3. Scatterplots relating resting state metrics and cognitive test performance

For ease of viewing, Figure 2 in the main text displays trend lines of our primary models without the data points. Data points underlying Figure 2 are plotted in Supplementary Figure 1, below. This figure illustrates the extent of individual variability in the relation, and the sheer number of participants.

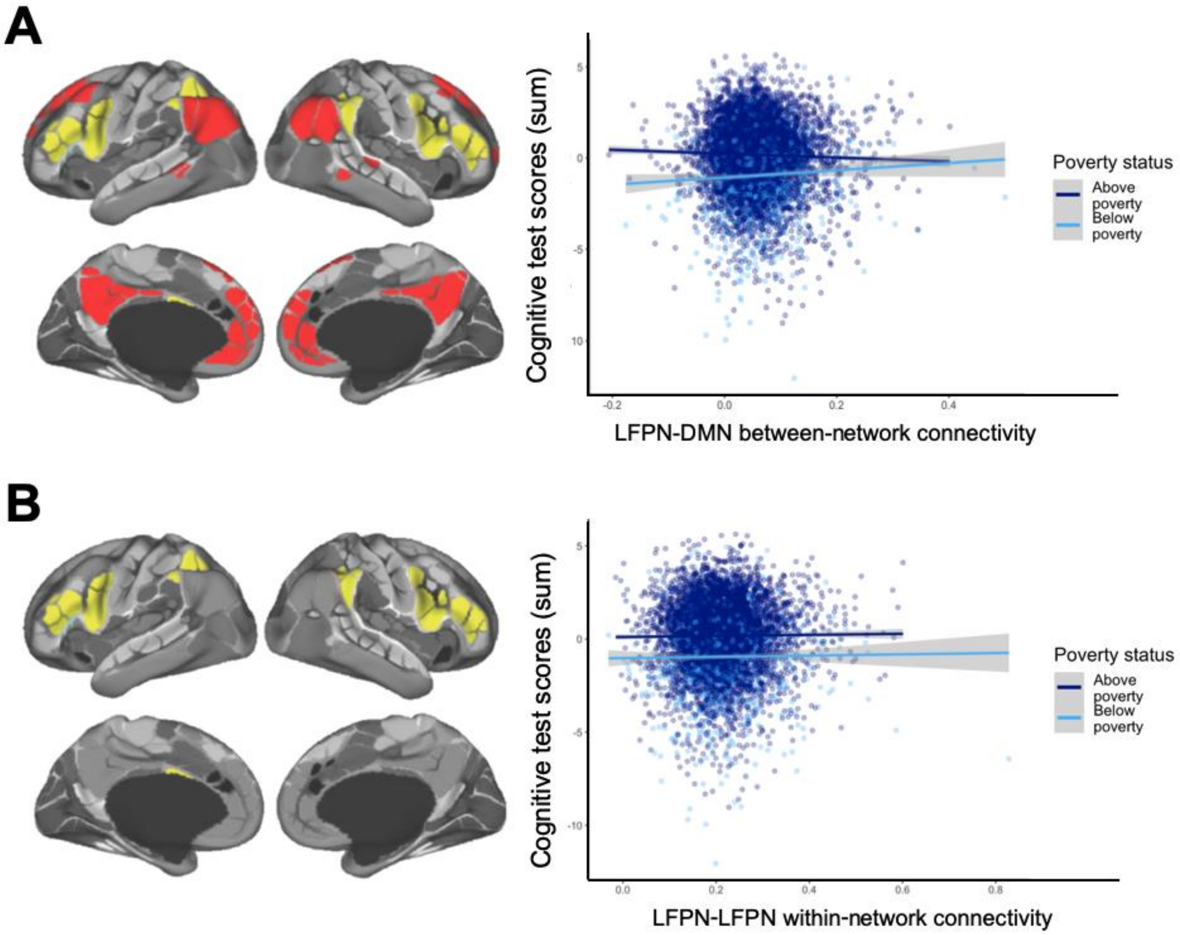

Supplementary Figure 1. Scatterplots with data points for relations between resting state network metrics and cognitive test score residuals, after accounting for fixed effects of age and motion and a random effect for study site, for children living above poverty (dark blue) and below poverty (light blue). Trend lines are presented as mean values +/-95% confidence intervals for a linear model, using the geom_smooth function in ggplot. Networks functionally defined using the Gordon parcellation scheme; lateral frontoparietal network (LFPN) shown in yellow, default mode network (DMN) shown in red; figures adapted from [1] and reprinted with permission from the authors.

Supplementary Note 4. Relations between LFPN-DMN connectivity and cognitive test performance, separated by test

Among the children in poverty, relations between LFPN-DMN connectivity and each cognitive test were in the positive direction, Matrix reasoning: $B = 1.12$, SE = 0.50, t (1028) = 2.25; χ^2 (1) = 5.07, $p = 0.024$; Flanker: $B = 0.18$, SE = 0.56, t (1028) = 0.32; χ^2 (1) = 0.1, $p = 0.751$; Dimensional Change Card Sort (DCCS) task: $B =$ 0.81, SE = 0.51, t (1028) = 1.6; χ^2 (1) = 2.57, $p = 0.109$.

In addition, mirroring our main results, this relation interacted with poverty status, reaching significance for reasoning, χ^2 (1) = 6.76, $p = 0.009$, and dimensional card sort, χ^2 (1) = 6.44, $p = 0.011$, but not for Flanker, χ^2 (1) = 1.42, $p = 0.233$.

We also repeated analyses using the NIH Toolbox Fluid Cognition composite, which includes two tests of working memory (Picture Sequence Memory Test, List Sorting Working Memory Test) and a test of processing speed (Pattern Comparison Processing Speed Test), in addition to Flanker and Dimensional Card Sort. (Note that Matrix Reasoning is not included in the fluid ability composite.)

Mirroring our primary results, relations between LFPN-DMN connectivity and the NIH Toolbox fluid ability composite were in the positive direction for children in poverty, $B =$ 9.19, SE = 4.95, t (1020) = 1.86; χ^2 (1) = 3.44, $p = 0.064$, and negative for children above poverty, $B = -8.55$, SE = 2.23, t (5766) = -3.84; χ^2 (1) = 14.7, $p < 0.001$. Likewise, the relation between LFPN-DMN connectivity and the NIH Toolbox fluid ability composite interacted significantly with poverty status, χ^2 (1) = 10.91, $p = 0.001$, as shown in Supplementary Figure 2 below.

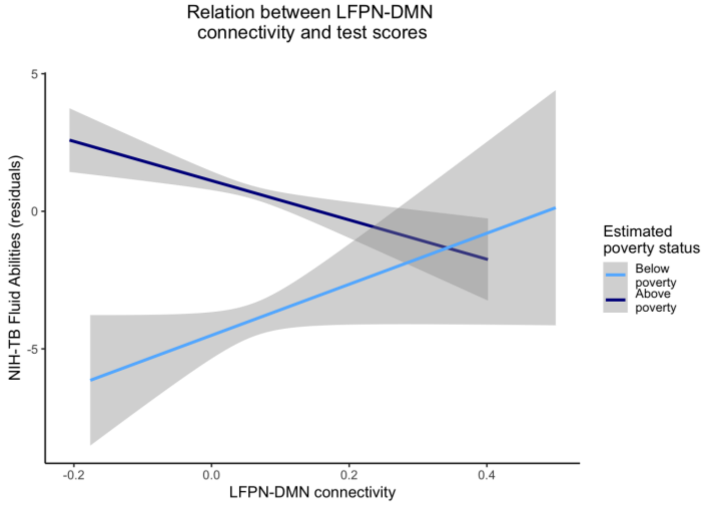

Supplementary Figure 2. Relations between lateral frontoparietal-default mode network (LFPN-DMN) connectivity and NIH-TB fluid abilities composite score residuals, for children living above poverty (dark blue) and below poverty (light blue). Models include fixed effects for age and motion and a random effect for study site. Data are presented as mean values +/-95% confidence intervals for a linear model, calculated and displayed using the geom_smooth function in ggplot.

Supplementary Note 5. Relations between LFPN-LFPN connectivity and cognitive test performance, separated by test

Among the children in poverty, the direction of association between LFPN-LFPN connectivity and each cognitive test were inconsistent, matrix reasoning: *B* = 0.69, SE = 0.38, *t* (1028) = 1.81; χ^2 (1) = 3.27, *p* = 0.070; Flanker: *B* = -0.44, SE = 0.42, *t* (1028) = -1.04; χ^2 (1) = 1.06, *p* = 0.303; dimensional card sort: *B* = -0.11, SE = 0.39, *t* (1028) = -0.28; χ^2 (1) = 0.08, *p* = 0.776.

There were no significant interactions between poverty status and LFPN-LFPN connectivity in predicting cognitive test scores, matrix reasoning: χ^2 (1) = 2.36, *p* = 0.125; Flanker: χ^2 (1) = 0.78, *p* = 0.376; dimensional card sort: χ^2 (1) = 0.56, *p* = 0.455.

Supplementary Note 6. Bootstrapped distribution of LFPN-DMN connectivity ~ test performance parameter estimates

Our first test was designed to probe how frequently the parameter estimate observed in the children in poverty would be expected to be observed in a larger sample of children living above poverty. In order to derive an estimate for observed parameter estimates in a population of higher-income children, we randomly sampled 500 data points from the children living above poverty, with replacement. For these 500 data points, we fit our primary linear mixed effects model to the data, predicting children's cognitive test scores, and calculated the average parameter estimate for LFPN-DMN connectivity. We repeated this process 999 times, generating a distribution of parameter estimates likely within the larger population of higher-income children from which our participants were drawn.

Next, we compared these bootstrapped parameter estimates to the parameter estimate observed for children in poverty in our sample. If the brain-behavior relation does not differ systematically as a function of poverty status—in other words, if the observed relation between LFPN-DMN connectivity and cognitive test scores for the children in poverty would be likely to be observed in a larger, population-level sample of children above poverty—the parameter estimate for children in poverty should fall within the 95% confidence interval of the bootstrapped parameter estimates.

Thus, we estimated the expected distribution of LFPN-DMN coefficients for the prediction of cognitive test scores among the higher-income children in the dataset. The results of this analysis confirmed that the observed estimate for children in poverty fell outside of the 95% CI, and was higher than 987 out of 999 bootstrapped samples, *p* = 0.013.

Repeating this bootstrapping procedure for children living below poverty revealed a similar effect. Bootstrapped coefficients ranged from -2.87 to 7.66, with a mean of 2.26, 95% CI [2.16, 2.35]. The observed estimate for children above poverty fell outside of the 95% CI, and was lower than 990 out of 999 bootstrapped estimates, *p* = 0.010.

The bootstrapped distributions from the two samples are plotted side by side in Supplementary Figure 3.

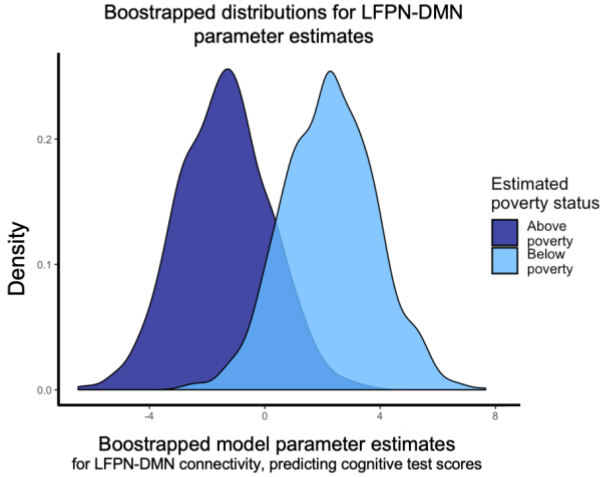

Supplementary Figure 3. Bootstrapped distributions for lateral frontoparietal-default mode network (LFPN-DMN) connectivity parameter estimates in the models predicting cognitive test performance, for children above (dark blue) and below (light blue) poverty. Estimates calculated from models run on 500 data points drawn with replacement from each sample separately, repeated 999 times. Bootstrapped coefficients for children above poverty ranged from -6.47 to 3.74, with a mean of -1.41, 95% CI [-1.50, -1.31], mirroring our observed parameter estimate for the higher-income group. The observed estimate for children in poverty fell outside of the 95% CI, and was higher than 987 out of 999 bootstrapped samples, $p = 0.013$. *P*-value caculated based on the number of times the estimate was higher for the children in poverty than for the bootstrapped distribution, divided by the number of bootstrapped observations plus one.

Supplementary Note 7. Permutation testing of LFPN-DMN connectivity ~ test performance parameter estimates

To further confirm the dissociation with LFPN-DMN connectivity and test performance for children living above or below poverty, we performed a permutation procedure. This procedure examined the extent to which the model parameters fit in the higher-income children alone could explain the data in the children in poverty.

Briefly, we used the model parameters generated from the higher-income children to predict test performance in the children in poverty, and calculated the mean difference (observed values minus predicted values). We next randomly permuted the labels of each group, such that assignment into the higher- versus lower-income group was now arbitrary. We repeated the process above, now fitting model parameters to our arbitrary higher-income group and using them to predict cognitive test performance in our arbitrary lower-income group. Again, we calculated the mean error between observed and predicted values. This permutation and prediction procedure was repeated 999 times, generating a distribution of mean differences when the distinction between the two groups was arbitrary. If the model parameters generated from our actual higher-income group could reasonably be applied to our actual lower-income group, we would expect that the mean error would fall within the 95% confidence interval of our distribution of permuted mean errors.

The results of this permutation procedure revealed that the model parameters fit on the children above poverty over-estimated the performance of children below poverty, on average (mean difference between observed and predicted test scores = -1.23). To contextualize whether this difference in prediction was larger than what would be expected by chance, we compared this to the distribution of 999 randomly group permuted labels, such that assignment into the higher- versus lower-income group was now arbitrary. The mean difference between the actual groups fell above all differences in the 999 permutations (range = -0.22 – 0.22), suggesting this difference is larger than would be expected by chance.

Supplementary Note 8. Relations between LFPN-DMN connectivity and cognitive test performance, for children with low thresholds of motion

Head motion, which is known to influence functional connectivity estimates (Power et al., 2015), differed significantly as a function of poverty status (see Table 1) and was correlated with cognitive test performance (B = -1.91, SE = 0.16, p < 0.001). Our reported analyses use a stringent motion exclusion criteria, in which participants were retained only if they had at least 12.5 minutes of data with low head motion (FD < 0.2 mm). Additionally, there was stringent motion correction in the analysis pipeline, as reported in the main text.

Still, we repeated these analyses with only those children who met a highly stringent motion criterion of less than or equal to 0.2 mm of average framewise displacement (N = 4444; 589 below poverty). Specifically, we fit a linear mixed effects model with site as a repeated measure, testing the relation between cognitive test scores and LFPN-DMN connectivity, controlling for age and head motion. In this subsample of participants who met our threshold for low motion (N = 4444; 589 below poverty), results were consistent, if not stronger. Specifically, for children living below poverty, the main effect of LFPN-DMN connectivity on test scores was positive and significant, B = 4.92, SE = 1.92, t (583) = 2.57; χ^2 (1) = 6.61, p = 0.010. Children living above poverty, in contrast, showed a negative main effect of LFPN-DMN connectivity, B = -1.27, SE = 0.62, t (3844) = -2.039; χ^2 (1) = 4.15, p = 0.041. The interaction between poverty status and LFPN-DMN connectivity was significant, χ^2 (1) = 11.93, p = 0.001 (consistent with the interaction effect in the full sample, χ^2 (1) = 8.99, p = 0.003). Thus, results were consistent—and seem to be even stronger—in this subsample of low-motion children.

Supplementary Note 9. Relations between LFPN-DMN connectivity and cognitive test performance, controlling for number of usable frames

A related concern is that our finding was driven by group differences in the number of usable frames of resting state data. Indeed, resting state metrics become more stable with more data[2]. In our data, the number of frames participants contributed after outliers were excluded ranged from 376-2170. We also found that LFPN-DMN connectivity was related to participants' number of usable frames, even when controlling

for mean framewise displacement, χ^2 (1) = 21.23, p < 0.001. However, frames of usable data no longer contributed to model fit when considering participants with relatively more usable frames (top 75% of usable frames, >759: χ^2 (1) = 2.03, p = 0.154; top 50% of usable frames, >1005: χ^2 (1) = 1.34, p = 0.247; top 25% of usable frames, >1199: χ^2 (1) = 1.36, p = 0.244).

To address whether scan length affected our results, we first reran our primary model testing the interaction between LFPN-DMN and poverty status in predicting cognitive test scores, with the additional covariate of number of usable frames after outliers were removed. The interaction between LFPN-DMN and poverty status remained significant, B = 3.14, SE = 1.06, t (6825) = 2.97, χ^2 (1) = 8.81, p = 0.003, and the number of usable frames did not contribute to model fit above and beyond framewise displacement, χ^2 (1) = 1.91, p = 0.167. (Framewise displacement continued to contribute significantly to model fit, χ^2 (1) = 20.43, p < 0.001.) Moreover, the interactive effect remained when restricting analyses to only those participants in the top 75th, 50th and 25th percentiles of usable frames (see Supplementary Figure 4 below for results and associated Ns).

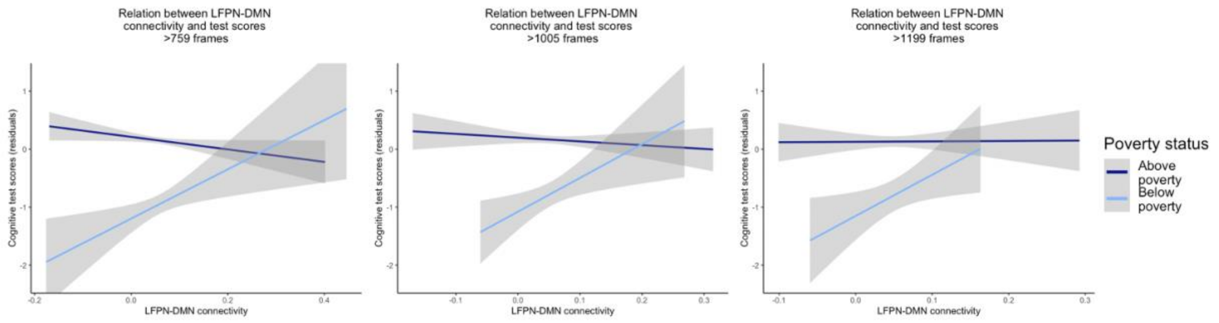

Supplementary Figure 4.
Interactions between lateral frontoparietal-default mode network (LFPN-DMN) connectivity and poverty status (children estimated to be living above poverty, dark blue, and below poverty, light blue) in predicting cognitive test performance. Mixed models include fixed effects for age and motion and a random effect for study site. Left: children with greater than 759 frames after outliers have been excluded, representing 75% of the sample (N = 5126 (700 below; 4426 above); Interaction: B = 5.45, SE = 1.44, t (5113) = 3.78; X^2 (1) = 14.25, p < .001). Center: children with greater than 1005 frames after outliers have been excluded, representing 50% of the sample (N = 3418 (439 below; 2979 above); Interaction: B = 6.95, SE = 2.04, t (3407) = 3.41; X^2 (1) = 11.6, p = .001). Right: children with greater than 1199 frames after outliers have been excluded, representing 25% of the sample (N = 1709 (215 below; 1494 above; Interaction: B = 7.98, SE = 3.0, t (1698) = 2.57; X^2 (1) = 6.56, p = .010). Data are presented as mean values +/-95% confidence intervals for a linear model, calculated and displayed using the geom_smooth function in ggplot.

Supplementary Note 10. Relations between LFPN-DMN connectivity and age

Given prior evidence that the LFPN and DMN become less correlated during childhood, we asked whether there was an effect of age in the current study. Indeed, even within this very restricted age range, LFPN-DMN connectivity was lower among

older children across the entire sample, B = -0.0003, SE = 0.0001, t (6832) = -2.93 p = 0.003. This pattern was consistent both for children below poverty, B = -0.0004, SE = 0.0003, t (1032) = -1.66, p = 0.097, and those above, B = -0.0002, SE = 0.0001, t (5798) = -2.40, p = 0.016 (Supplementary Figure 4). We note that, while children in poverty had marginally higher LFPN-DMN connectivity overall (see Table 1), children living above poverty were approximately 17 days older than children living below poverty. The difference in connectivity between these groups was eliminated when accounting for this slight age difference (see Table 1).

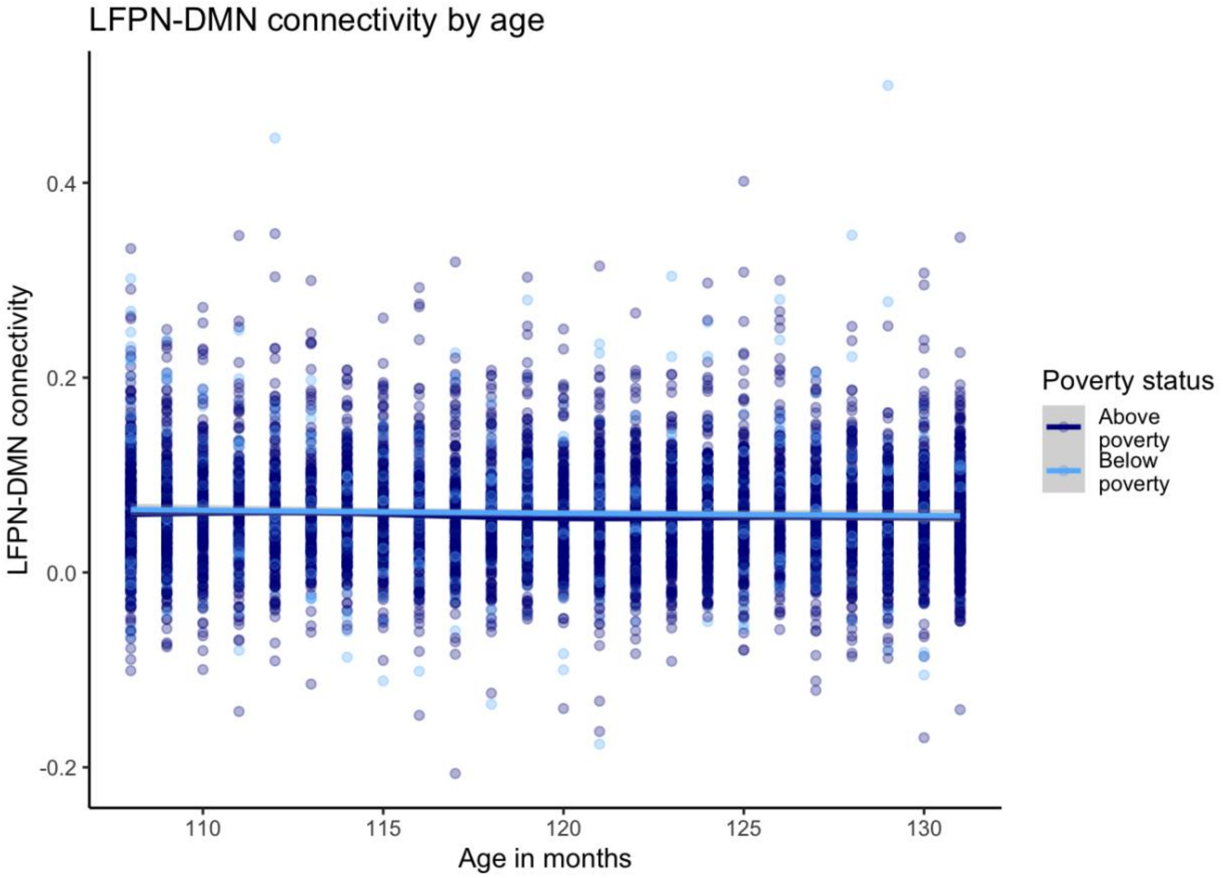

Supplementary Figure 5.
Relations between lateral frontoparietal-default mode network (LFPN-DMN) connectivity and child age, for children estimated to be living above poverty (dark blue) and below poverty (light blue).

Supplementary Note 11. Ridge regression confidence intervals

We randomly divided our test set and training set and found that it predicted approximately 4% (R^2_{CV} = 0.037) of the variance in test performance. However, one concern is that this split of the data happened to be particularly lucky, and unrepresentative of other possible splits. Therefore, we repeated this random split 1,000 times, training a new model on a randomly selected two-thirds of children and testing it in the remaining one-third of held out children. Thus, we were able to calculate a distribution of R^2 values. This repeated subsampling revealed that the model trained in

two-thirds of the children in poverty predicted the held-out sample of children in poverty at above chance levels in more than 95% of iterations (in a cross-validation framework, this means $R^2 > 0$). The mean of this distribution was 0.023, 95% CI [0.021, 0.024]. Thus, while our split and model was on the high end of possible model fits, the model did consistently perform above chance.

Supplementary Note 12. Deviations from pre-registration

Both pre-registrations were written before knowing which data from the ABCD study would be available for analysis, which led to some necessary changes upon receipt of the data. In our first pre-registration, we planned to examine the relation between reasoning performance and specific node-to-node connectivity; however, only summary network measures had been released when we conducted our investigation. We also planned to look at test scores longitudinally, but found that only the first timepoint of cognitive assessments had been completed. Thus, we focused our analyses on one of our two primary planned questions. In addition, we planned to run simple linear regressions; these did not take into account the nested structure of the data, which we ultimately addressed in a data-driven fashion using linear mixed effects models, as described in the analysis section of the main text. The nested structure of the data also made our planned cross-validation approach less feasible, and we therefore did not cross-validate this first set of analyses. Finally, we planned to define our poverty threshold based on the Supplemental Poverty Threshold for each study site; however, due to privacy issues with de-identifying study site, we were only able to use a coarser threshold averaging across study sites. In our second pre-registration, we listed three environmental variables that were not collected at the baseline visit: self-reported discrimination, negative life events, and positive life events. Finally, we made the decision to include ethnicity separate from race, as it was collected, to retain maximal information. Otherwise, all analyses were performed as planned.

Several previously unspecified decisions were also made in the analysis process. First, we chose to use raw, rather than age-standardized, cognitive test scores. The rationale was that for using raw scores was that (1) the age range within our sample is relatively tight, (2) brain-behavior relations are of interest within the sample, not in relation to test norms based on a different sample of children, and (3) brain imaging data we are using aren’t age normalized. Second, several factor levels within the environmental variables had a very low incidence in the whole sample, with less than 15 participants total (school setting: cyber school; school setting: vocational/tech school; race/ethnicity: Native Hawaiian); these were grouped into the “other” designation for the given factor, to allow for successful cross-validation when the sample was split further. Third, we made the decision to impute missing data from the environmental variables to preserve sample size.

Supplementary Note 13. References

1. Gordon, E. M. *et al.* Generation and Evaluation of a Cortical Area Parcellation from Resting-State Correlations. *Cereb. Cortex* **26**, 288–303 (2016).
2. Gordon, E. M. *et al.* Precision Functional Mapping of Individual Human Brains. *Neuron* **95**, 791-807.e7 (2017).
