# Supplementary material for: Brain network coupling associated with cognitive performance varies as a function of a child’s environment in the ABCD study
Source: Nat Commun. 2021 Dec 10;12:7183. doi: 10.1038/s41467-021-27336-y (PMC8664837; doi:10.1038/s41467-021-27336-y)
Supplement: Supplementary file 3 — Reporting summary [file 41467_2021_27336_MOESM3_ESM.pdf]

nature research

Corresponding author(s): Monica E Ellwood-Lowe
Last updated by author(s): Sep 28, 2021

Reporting Summary

Nature Research wishes to improve the reproducibility of the work that we publish. This form provides structure for consistency and transparency in reporting. For further information on Nature Research policies, see our Editorial Policies and the Editorial Policy Checklist.

Statistics

For all statistical analyses, confirm that the following items are present in the figure legend, table legend, main text, or Methods section.

n/a	Confirmed	
☐	☒	The exact sample size (*n*) for each experimental group/condition, given as a discrete number and unit of measurement
☐	☒	A statement on whether measurements were taken from distinct samples or whether the same sample was measured repeatedly
☐	☒	The statistical test(s) used AND whether they are one- or two-sided<br>*Only common tests should be described solely by name; describe more complex techniques in the Methods section.*
☐	☒	A description of all covariates tested
☐	☒	A description of any assumptions or corrections, such as tests of normality and adjustment for multiple comparisons
☐	☒	A full description of the statistical parameters including central tendency (e.g. means) or other basic estimates (e.g. regression coefficient) AND variation (e.g. standard deviation) or associated estimates of uncertainty (e.g. confidence intervals)
☐	☒	For null hypothesis testing, the test statistic (e.g. *F*, *t*, *r*) with confidence intervals, effect sizes, degrees of freedom and *P* value noted<br>*Give P values as exact values whenever suitable.*
☒	☐	For Bayesian analysis, information on the choice of priors and Markov chain Monte Carlo settings
☒	☐	For hierarchical and complex designs, identification of the appropriate level for tests and full reporting of outcomes
☐	☒	Estimates of effect sizes (e.g. Cohen's *d*, Pearson's *r*), indicating how they were calculated

Our web collection on statistics for biologists contains articles on many of the points above.

Software and code

Policy information about availability of computer code

Data collection	Data came from the larger, ongoing Adolescent Brain Cognitive Development (ABCD) study. Full information on data collection procedures can be found at http://abcdstudy.org. Parents and children filled out demographic surveys and children completed cognitive tests through the NIH Toolbox software and the WISC-V Matrix Reasoning subtest (iPad versions); children completed MRI scans on 3T scanners from which resting state metrics were derived.
Data analysis	Analysis plans were pre-registered prior to data access (https://aspredicted.org/blind.php?x=3d7ry9, https://aspredicted.org/blind.php?x=tg4tg9) and analysis scripts are openly available on the Open Science Framework (https://osf.io/hs7cg/?view_only=d2acb721549d4f22b5eeea4ce51195c7). Analyses were performed using R version 3.6.0 (R Core Team, 2017).<br><br>R packages used for data cleaning, analysis, and visualization include: dplyr (Wickham et al., 2019); ggplot2 (Wickham, 2016); car (J. Fox & Weisberg, 2011); corrplot (Wei & Simko, 2017); MuMIn (Bartoń, 2019); tidyr (Wickham & Henry, 2019); summarytools (Comtois, 2019); finalfit (Harrison et al., 2019); fastDummies (Kaplan, 2019); caret (from Jed Wing et al., 2019); scales (Wickham, 2018); foreign (R Core Team, 2018); MASS (Venables & Ripley, 2002); sjPlot (Lüdecke, 2019); tableone (Yoshida, 2019); gtools (Warnes et al., 2018); ridge (Cule & Moritz, 2019); robustlmm (Koller, 2016); lavaan (Rosseel, 2012); glmnet (Friedman et al., 2010); mice (van Buuren & Groothuis-Oudshoorn, 2011); lme4 (D. Bates et al., 2015).

For manuscripts utilizing custom algorithms or software that are central to the research but not yet described in published literature, software must be made available to editors and reviewers. We strongly encourage code deposition in a community repository (e.g. GitHub). See the Nature Research guidelines for submitting code & software for further information.

Data

Policy information about availability of data

All manuscripts must include a data availability statement. This statement should provide the following information, where applicable:

- Accession codes, unique identifiers, or web links for publicly available datasets
- A list of figures that have associated raw data
- A description of any restrictions on data availability

All raw and processed data used for these analyses are available in the ABCD Data Repository in the National Institute of Mental Health (NIMH) Data Archive Collection #2573 (https://nda.nih.gov/abcd). To obtain permission to these data, users must create an account through the NIMH Data Archive and follow the instructions on the website to gain access.

Field-specific reporting

Please select the one below that is the best fit for your research. If you are not sure, read the appropriate sections before making your selection.

☐ Life sciences ☒ Behavioural & social sciences ☐ Ecological, evolutionary & environmental sciences

For a reference copy of the document with all sections, see nature.com/documents/nr-reporting-summary-flat.pdf

Behavioural & social sciences study design

All studies must disclose on these points even when the disclosure is negative.

Study description	The current study uses an observational quantitative cross-sectional approach on the first wave of a longitudinal study.
Research sample	Participants were selected from the larger, ongoing Adolescent Brain Cognitive Development (ABCD) study, which was designed to recruit a cohort of children who closely represented the United States population (http://abcdstudy.org; see Garavan et al., 2018). We analyzed the data from 6839 children ages 9-10 years based on the sampling strategy and exclusion criteria listed below, approximately 50% of whom identified as female, and 50% as male. Our primary analyses focused on 1034 children whose families made less than $25,000 for families of 4 and less, or less than $35,000 for families of 5 and more. Additional sample characteristics are reported in the demographic table of the main text. This sample was chosen because it is the most demographically representative of the US national population of adolescent neuroimaging to date.
Sampling strategy	The sampling strategy for the larger, ongoing study is reported in Garavan et al., 2018. For our current study, of all the available data from the first wave of the ABCD study, we excluded children who did not provide information about family income and complete data on all three cognitive tests, and/or if their MRI data did not meet ABCD's usability criteria (see below). In addition, due to a scanner error, we excluded post-hoc all children who were scanned on Philips scanners. This left us with 1034 children identified as likely to be living below poverty (6839 across the whole sample).
Data collection	Data were collected at each separate study site, as described further in Garavan et al., 2018. Children completed neurocognitive batteries on an iPad when parents were not present (described above and in Luciana et al., 2018) and parents filled out demographic information using REDCap; children also completed MRI scans, with scanning procedures described in Casey et al., 2018. Researchers collecting the data were not aware of the hypotheses for the current study.
Timing	Data used in the current study were collected between September 2016 and October 2018.
Data exclusions	Exclusion criteria were determined ahead of time and pre-registered. Of the 11,875 children who completed the baseline session, we excluded children who did not provide information about family income and complete data on all three cognitive tests, and/or if their MRI data did not meet ABCD's usability criteria (see below). In addition, due to a scanner error, we excluded post-hoc all children who were scanned on Philips scanners. This left us with 1034 children identified as likely to be living below poverty (6839 across the whole sample).
Non-participation	This information from the larger ABCD study is not available to us.
Randomization	No groups.

Reporting for specific materials, systems and methods

We require information from authors about some types of materials, experimental systems and methods used in many studies. Here, indicate whether each material, system or method listed is relevant to your study. If you are not sure if a list item applies to your research, read the appropriate section before selecting a response.

Materials & experimental systems

n/a	Involved in the study
☒	☐ Antibodies
☒	☐ Eukaryotic cell lines
☒	☐ Palaeontology and archaeology
☒	☐ Animals and other organisms
☐	☒ Human research participants
☒	☐ Clinical data
☒	☐ Dual use research of concern

Methods

n/a	Involved in the study
☒	☐ ChIP-seq
☒	☐ Flow cytometry
☐	☒ MRI-based neuroimaging

Human research participants

Policy information about studies involving human research participants

Population characteristics	See above.
Recruitment	Participants were recruited through schools with dynamic monitoring of population demographics in order to reduce selection bias as much as possible. This procedure is described fully in Garavan et al., 2018.
Ethics oversight	This study was approved by the Institutional Review Board at each study site, with centralized IRB approval from the University of California, San Diego.

Note that full information on the approval of the study protocol must also be provided in the manuscript.

Magnetic resonance imaging

Experimental design

Design type	Resting state; t1-weighted scan
Design specifications	After completing motion compliance training in a simulated scanning environment, participants first completed a structural T1-weighted scan. Next, they completed three to four five-minute resting state scans, in which they were instructed to lay with their eyes open while viewing a crosshair on the screen. The first two resting state scans were completed immediately following the T1-weighted scan; children then completed two other structural scans, followed by one or two more resting state scans, depending on the protocol at each specific study site.
Behavioral performance measures	Behavioral performance measures are not collected during administration of these scans.

Acquisition

Imaging type(s)	Functional, structural
Field strength	3 Tesla
Sequence & imaging parameters	Scan parameters were optimized to be compatible across scanner platforms, allowing for maximal comparability across the 19 study sites. All T1-weighted scans were collected in the axial position, with 1mm3 voxel resolution, 256 x 256 matrix, 8 degree flip angle, and 2x parallel imaging. Other scan parameters varied by scanner platform (Siemens: 176 slices, 256 x 256 FOV, 2500 ms TR, 2.88 ms TE, 1060 ms TI; Philips: 225 slices, 256 x 240 FOV, 6.31 ms TR, 2.9 ms TE, 1060 ms TI; GE: 208 slices, 256 x 256 FOV, 2500 ms TR, 2 ms TE, 1060 ms TI). All fMRI scans were collected in the axial position, with 2.4mm3 voxel resolution, 60 slices, 90 x 90 matrix, 216 x 216 FOV, 800ms TR, 30 ms TE, 52 degree flip angle, and 6 factor MultiBand Acceleration. Motion was monitored during scan acquisition using real-time procedures to adjust scanning procedures as necessary (see Casey et al., 2018).
Area of acquisition	Whole brain

Diffusion MRI ☐ Used ☒ Not used

Preprocessing

Preprocessing software	Data processing was carried out using the ABCD pipeline and carried out by the ABCD Data Analysis and Informatics Core; more details are reported by Hagler et al. (2019).
Normalization	T1-weighted images were corrected for gradient nonlinearity distortion and intensity inhomogeneity, and rigidly registered to a custom atlas. For resting state images, after initial scan volumes were removed, each voxel was normalized and demeaned.
Normalization template	Custom atlas
Noise and artifact removal	Resting state images were first corrected for head motion, displacement estimated from field map scans, B0 distortions, and

gradient nonlinearity distortions, and registered to the structural images using mutual information. Signal from motion, quadratic trends, and mean time courses of white matter, gray matter, and whole brain, plus first derivatives, were regressed out. The data underwent temporal bandpass filtering (0.009 – 0.08 Hz).

Volume censoring

Initial scan volumes were removed, and subsequent frames with greater than 0.3mm displacement were excluded.

Statistical modeling & inference

Model type and settings

Filtered and corrected time courses were projected onto FreeSurfer's cortical surface, upon which 13 functionally-defined networks (Gordon et al., 2016) were mapped and time courses for FreeSurfer's standard cortical and subcortical ROIs extracted (Desikan et al., 2006; Fischl et al., 2002). Correlations for each pair of ROIs both within and across each of the 13 networks were calculated. These were z-transformed and averaged to calculate within-network connectivity for each network (the average correlation of each ROI pair within the network) and between-network connectivity across all networks (the average correlation of pairs of each ROI in one network with each ROI in another network).

Effect(s) tested

Here, we examined only within-network connectivity for LFPN and between-network LFPN-DMN connectivity, using the estimates derived above.

Specify type of analysis: ☐ Whole brain ☒ ROI-based ☐ Both

Anatomical location(s)

Using the Gordon parcellation networks (Gordon et al., 2016)

Statistic type for inference
(See Eklund et al. 2016)

Voxel-wise or cluster-wise analyses were not performed.

Correction

Voxel-wise or cluster-wise analyses were not performed.

Models & analysis

n/a	Involved in the study
☐	☒ Functional and/or effective connectivity
☒	☐ Graph analysis
☒	☐ Multivariate modeling or predictive analysis

Functional and/or effective connectivity

Average correlation of each ROI pair within the network(s) of interest, calculated as described above.
